# Reaction Kinetics for the Biocatalytic Conversion of Phenazine-1-Carboxylic Acid to 2-Hydroxyphenazine

**DOI:** 10.1371/journal.pone.0098537

**Published:** 2014-06-06

**Authors:** Mingmin Chen, Hongxia Cao, Huasong Peng, Hongbo Hu, Wei Wang, Xuehong Zhang

**Affiliations:** State Key Laboratory of Microbial Metabolism, School of Life Sciences and Biotechnology, Shanghai Jiao Tong University, Shanghai, People's Republic of China; Instituto de Biociencias - Universidade de São Paulo, Brazil

## Abstract

The phenazine derivative 2-hydroxyphenazine (2-OH-PHZ) plays an important role in the biocontrol of plant diseases, and exhibits stronger bacteriostatic and fungistatic activity than phenazine-1-carboxylic acid (PCA) toward some pathogens. PhzO has been shown to be responsible for the conversion of PCA to 2-OH-PHZ, however the kinetics of the reaction have not been systematically studied. Further, the yield of 2-OH-PHZ in fermentation culture is quite low and enhancement in our understanding of the reaction kinetics may contribute to improvements in large-scale, high-yield production of 2-OH-PHZ for biological control and other applications. In this study we confirmed previous reports that free PCA is converted to 2-hydroxy-phenazine-1-carboxylic acid (2-OH-PCA) by the action of a single enzyme PhzO, and particularly demonstrate that this reaction is dependent on NADP(H) and Fe^3+^. Fe^3+^ enhanced the conversion from PCA to 2-OH-PHZ and 28°C was a optimum temperature for the conversion. However, PCA added in excess to the culture inhibited the production of 2-OH-PHZ. 2-OH-PCA was extracted and purified from the broth, and it was confirmed that the decarboxylation of 2-OH-PCA could occur without the involvement of any enzyme. A kinetic analysis of the conversion of 2-OH-PCA to 2-OH-PHZ in the absence of enzyme and under different temperatures and pHs *in vitro*, revealed that the conversion followed first-order reaction kinetics. In the fermentation, the concentration of 2-OH-PCA increased to about 90 mg/L within a red precipitate fraction, as compared to 37 mg/L within the supernatant. The results of this study elucidate the reaction kinetics involved in the biosynthesis of 2-OH-PHZ and provide insights into *in vitro* methods to enhance yields of 2-OH-PHZ.

## Introduction

Phenazine compounds are of interest because of their broad spectrum activity against soil born root disease [1], [2]. They encompass a large family of natural heterocyclic nitrogen-containing compounds that are produced in late exponential and stationary growth phase of some strains. Over 100 natural phenazine compounds with the same basic structure are known, differing only in the derivatization of the heterocyclic core. These differences largely determine the physical properties of the phenazines and greatly influence their biological activity toward plant and animal pathogens. Natural phenazine derivatives are synthesized primarily by *Pseudomonas spp.* and *Streptomyces spp.* [3], [4], especially *Pseudomonas chlororaphis* spp. [5], [6], [7]. The most commonly identified and evaluated phenazine derivatives produced by *P. chlororaphis spp.* are phenazine-1-carboxylic acid (PCA), phenazine carboxamide (PCN) and a number of hydroxy-phenazines [8].

*Pseudomonas chlororaphis* GP72 [9], a plant-beneficial rhizobacterium that has shown broad-spectrum antifungal activity against various phytopathogens of agricultural significance, produces three phenazine compounds [9], [10], [11]: PCA, 2-hydroxy-phenazine-1-carboxylic acid (2-OH-PCA) [11] and 2-hydroxy-phenazine (2-OH-PHZ) [12], [13]. 2-OH-PHZ is derived from PCA by the unique modification of a key enzyme PhzO which belongs to a family of two-component nonheme flavin-diffusible bacterial aromatic monooxygenases. This enzyme was first discovered in *Pseudomonas chlororaphis subsp. aureofaciens*30-84 [14], then also identified in *Pseudomonas chlororaphis* GP72 which shares 98% gene similarity with strain 30-84 [11].

Previous studies have shown that 2-hydroxyphenazines including 2-OH-PHZ and 2-OH-PCA exhibit stronger bacteriostatic and fungistatic activity compared with PCA toward some pathogens such as *Gaemannomyces. graminis* var. *tritici* [14], [15], [16]. 2-OH-PHZ is produced primarily by *P. chlororaphis* except for the strain *P. aurantiaca* PB-St2 [17].

The biosynthetic pathway leading to the production of 2-OH-PHZ was first described in *P. chlororaphis* 30–84 [18]. In this early study, it was shown that when *in trans*, a cosmid containing 30–84 genomic DNA comprising the coding sequence of only 5 of the 7 genes in what was later shown to be the phenazine biosynthetic operon was sufficient to produce all three phenazines at low level in *E.coli*. It was hypothesized that only a part of *phzC* was needed to convert PCA to 2-OH-PCA in *E.coli* [19]. In later work Mavrodi *et al.* [20] suggested that other *E.coli* enzymes likely contribute to the small amount of phenazines made by *E.coli* when only 5 genes were introduced; the amount of phenazine produced was substantially increased when the entire 7 gene operon now known to be important for PCA synthesis was present. Subsequently Delaney *et al.* [14] verified the entire biosynthetic pathway leading to 2-OH-PHZ. Importantly, they showed that 2-OH-PHZ was produced from PCA by the action of a single enzyme PhzO, which catalyzed the conversion of PCA to 2-OH-PCA, and that 2-OH-PCA was then spontaneously decarboxylated to form 2-OH-PHZ. Knowledge of this biosynthesis pathway provides the possibility to enhance production of 2-OH-PHZ for its application in agriculture [15], [21].

However, the yield of 2-OH-PHZ using chemical methods or via biosynthetic production in *Pseudomonas* spp. is relatively low [9], [16] in contrast with the production of its precursor, PCA [22], [23]. This limitation on production has become the main obstacle to widespread application of 2-OH-PHZ. Thus, it is important to conduct systematic studies and understand the reaction kinetics of 2-OH-PHZ and explore methods to produce 2-OH-PHZ in high yield.Herein, the catalytic conditions of PhzO were studied, 2-OH-PCA was extracted and purified from the broth, and the kinetics of the conversion of 2-OH-PCA to 2-OH-PHZ was studied systematically *in vitro*.

## Methods and Materials

### Bacterial strains and plasmid construction

Strains and plasmids are listed in Table 1. *Escherichia coli* BL21, *Pseudomonas chlororaphis* GP72 and its mutants [11] were obtained from our lab stock preserved in 20% (vol/vol) glycerol at −70°C. Unless indicated otherwise, *E. coli* was routinely grown at 37°C in Luria-Bertani (LB) medium. *P. chlororaphis* GP72 and its mutants were incubated at 28°C in LB and King's B (KB) broth, respectively. LB medium supplemented with 50 µg/ml kanamycin was used for *phzO* gene over-expression.

**Table 1:** List of bacterial strains, PCR products, primers and plasmids used in this study.

Strains	Characteristics	Reference
*E. coli* BL21	*recA*1 *endA*1 *gyrA*96 *thi*1 *hsdR*17 (*rk^−^ mk^+^*) *supE*44 *re1A*1	Sambrook & Rossel
*P. chlororaphis* GP72	PCA, 2-OH-PHZ producer	Liu *et al.*
*P. chlororaphis* GP72AN	PCA, 2-OH-PCA, 2-OH-PHZ producer, Gm^ r^	Huang *et al.*
*P. chlororaphis* GP72ON	PCA producer, Cm^ r^	Huang *et al*
*P. chlororaphis* GP72FN	No phenazine producer	Zhao *et al*
Plasmids	Characteristics	Source
pET28a	expression vector, *T7* promoter, 6-His tag, Kan^r^	Xuping lab
pET28a-phzO	1476-bp NdeI- BamHI PCR amplified fragment containing *phzO* cloned into pET28a	This study
Primers	Sequence (5' - 3')	Source
phzO-F(*Nde*I)	5′-CCCGAACATATGCTAGATCTTCAAAACAAGCGT-3′	This study
phzO-R(*BamH*I)	5′-TTTGGATCCCTATTTGGCGTTGAGCCCCACCA-3′	This study

### Cloning, expression, and purification of recombinant *phzO*

Plasmid construction: A pair of primers phzOF and phzOR was used (Table 1) to clone *phzO* at an annealing temperature of 58°C with the genome of strain GP72 as the template. The 1.5 kb PCR product was purified by agarose gel electrophoresis, and then digested with *EcoR*I and *Xho*I, the fragment was inserted into the multiple cloning site of pET28a, an expression vector, to obtain pET28a-*phzO*. Then the plasmid was introduced into *E. coli* BL21 to give *E. coli* BL21-*phzO* which was used for *phzO* gene over-expression.

For protein expression, the bacteria were cultured until the OD_600_ reached 0.4–0.6, and IPTG was added to a final concentration of 0.1 mM. After 8 h incubation, the bacteria were harvested and lysed by sonication in buffer A (20 mM Tris-HCl, 0.5 mM PMSF, 1 mM DTT). The lysate was cleared by centrifugation for 15 min at 10,000×g and then applied to a Ni-NTA-agarose column (Superflow Cartridge,QIAGEN) in buffer A**.** The recombinant PhzO was purified to homogeneity as previously described [24].

### PhzO activity assay *in vitro* and *in vivo*

PhzO activity assay *In vivo*: *E. coli* BL21 harboring pET28a or pET28a-phzO was grown at 37°C in LB broth supplemented with 50 µg/ml kanamycin for about 13 h, and then was diluted to fresh LB broth containing the same kanamycin concentration at a ratio of 1∶40. PCA was added to a final concentration of 0.3–0.7 mg/ml from a 25 mM stock solution in 55% (wt/vol) NaHCO_3_. The strain was cultivated at 37°C and 180 rpm to an optical density of 0.6 at 600 nm, and then induced with 0.1 mM IPTG. Samples were extracted, and analyzed for phenazine composition by high-performance liquid chromatography (HPLC).

PhzO activity assay *in vitro*: Crude cell extracts were prepared by sonication treatment of recombinant *E. coli* cells and assayed for hydroxyl activity by adding free PCA under different conditions. The effects of different metal ions were determined *in vitro* in 0.1 M Tris-HCl buffer (Table 2).

**Table 2:** Results of the *in vitro* PhzO activity assay.

	NAD(H)	NADP(H)	Co^2+^	Mn^2+^	Mg^2+^	Fe^3+^	PCA	2-OH-PCA	2-OH-PHZ
1	−	−	−	−	−	−	+	ND	ND
2	−	+	−	−	−	−	+	ND	ND
3	−	+	+	−	−	−	+	ND	ND
4	−	+	−	+	−	−	+	ND	ND
5	−	+	−	−	+	−	+	ND	ND
6	−	+	−	−	−	+	+	D	D
7	+	−	−	−	−	−	+	ND	ND
8	+	−	+	−	−	−	+	ND	ND
9	+	−	−	+	−	−	+	ND	ND
10	+	−	−	−	+	−	+	ND	ND
11	+	−	−	−	−	+	+	ND	ND
12	−	−	−	−	−	−	−	ND	ND

+ represents the addition of the specified metal ion, the final concentration of metal ion was 1 mM; the final concentration of PCA was 200 mg/L.

D, detectable.

ND, not detectable.

### The conversion of 2-OH-PCA to 2-OH-PHZ in cell extracts of *Pseudomonas*

To test the influence of enzymes in cell extracts of *Pseudomonas* on the reaction that the intermediate product 2-OH-PCA underwent spontaneous decarboxylation to form 2-OH-PHZ, 30 ul 100 mg/L purified 2-OH-PCA was added to 1 mL crude cell extracts of GP72FN and GP72ON at 28°C, respectively, and incubated for 18h in PBS (pH = 7.0). Crude cell extracts were prepared by sonication treatment of GP72FN and GP72ON after 24h incubation in KB at 28°C.

### Kinetics at different temperatures

The kinetic experiments were carried out in polypropylene tubes containing 1 mg purified 2-OH-PCA and 20 mL of phosphate-buffered saline solutions (PBS) at pH of 7.0. The experiments were performed in a waterbath at 28°C, 37°C, 55°C and 70°C from 0 to 23 h.

### Effect of initial pH

To evaluate the effect of pH on the spontaneous transformation of 2-OH-PCA to 2-OH-PHZ, 1 mg purified 2-OH-PCA was exposed to 10 mL of PBS in polypropylene tubes at pH from 3 to 11 at 70°C. The suspensions were shaken in a 20°C water bath for 5 min. For all of the experiments, concentrated NaOH and HCl solutions were used to adjust the pH of the mixtures.

### Effect of different irradiation

To evaluate the effect of different irradiation on the reaction of 2-OH-PCA to 2-OH-PHZ, 1 mg purified 2-OH-PCA was added to 20 mL of phosphate-buffered saline solutions (PBS) at pH of 7.0, and then exposed the different light sources at room temperature.

### Quantification of phenazine compounds

The fermentation broth was adjusted to pH 2.0 with 6 N HCl, before centrifugation at 10,000×g for 10 minutes using an Eppendorf Minispin centrifuge. Cellular debris was removed and the clear supernatant was collected and extracted with an equal volume of ethyl acetate with vigorously shaking [9],[11]. The collected organic layer was mixed with 1/10 volume distilled water and shaken rigorously. Finally, the organic phase containing 2-OH-PHZ was evaporated under vacuum pressure. The 2-OH-PHZ residue was dissolved in methanol for further analysis.

The HPLC analysis method for simultaneous analysis of the phenazine compounds PCA, 2-OH-PCA and 2-OH-PHZ was carried out using a 1260 Infinity HPLC apparatus (Agilent Technologies Group, Beijing, China)equipped with a UV detector and a C-18 reverse phase column (Agilent, USA) as described previously [11].

### Reagents

PCA (>95%), 2-OH-PCA (99.4% purity) and 2-OH-PHZ (99.0% purity) were prepared by our laboratory (Laboratory of Microbial Resources and Metabolic Engineering). The red pigment precipitate from the GP72 culture was suspended in water, and extracted using a double volume of acetic acid/ether, then evaporated by rotary evaporation. Further purification was performed by preparative liquid chromatography (Shimadzu LC-20AP) using a previously described method [11]. 2-OH-PCA (99.4%purity) was prepared and used for the following experiments. HPLC grade methanol (Lingfeng Chemical Reagent Co. Ltd., Shanghai, China) and ammonium acetate (Sigma Chemicals Co.) were used for the HPLC analysis, and all other chemicals were reagent grade (Sinopharm Chemical Reagent Co. Ltd., Shanghai, China). The pure water was produced by a water purifier (Aquapro DZG-303A; Zhongqin, China).

### Statistical analysis

Differences between treatments were determined by analysis of variance followed by Students test (P<0.05).

## Results

### Expression and purification of PhzO

Heterogenous expression of PhzO in *E.coli* BL21 was similar to the work done by Daleney *et al* [14]. PhzO was successfully expressed in *E.coli* BL21 (S1), and converted free PCA to 2-OH-PCA, then decarboxylated to form 2-OH-PHZ (S2). Other factors important for 2-OH-PHZ biosynthesis including different temperatures, IPTG concentrations and induction times of expression were examined, and the amount of PhzO in the soluble fraction and in inclusion bodies was studied by SDS-PAGE. Unfortunately, protein was detected in both soluble fraction and inclusion bodies under all conditions. The best results were achieved at 20°C after 8 h incubation induced with 0.1 mM IPTG as these conditions minimized the amount of PhzO found in inclusion bodies compared with 37°C.

**Figure 1:**
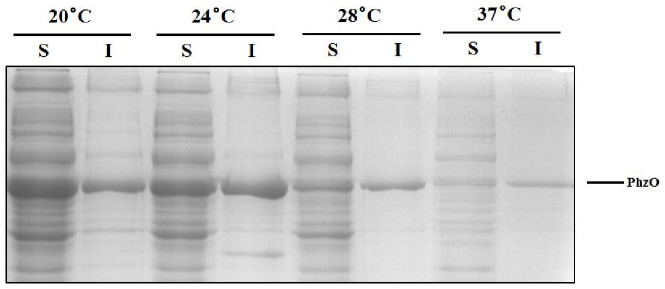
The expression profile of protein PhzO in *E.coli* BL21 at different temperatures. I represents **Insoluble** aggregates, S represents **Soluble** supernatants.

**Figure 2:**
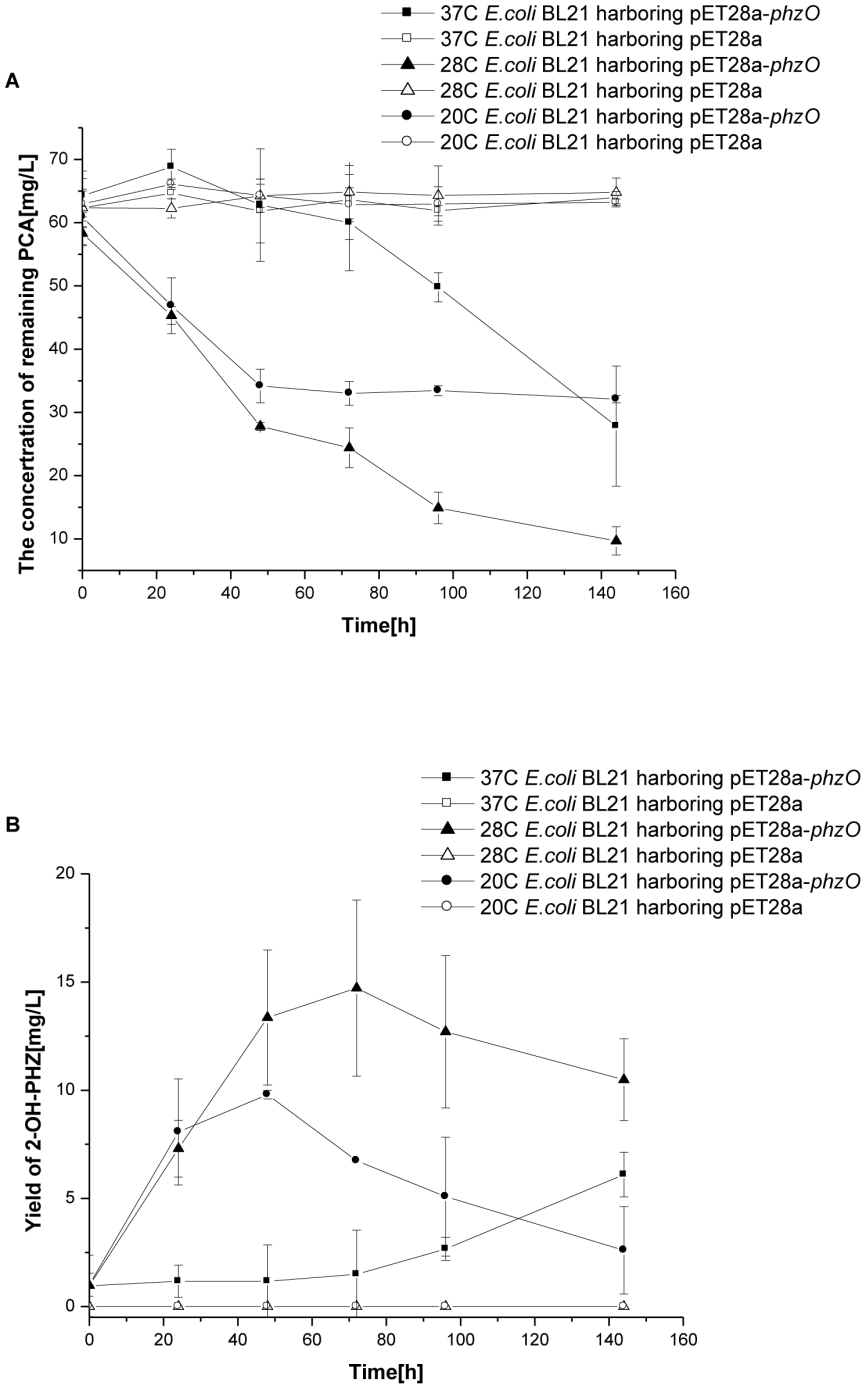
The conversion of PCA to 2-OH-PHZ at different temperatures. A Degradation of PCA at different temperature over time. B The production of 2-OH-PHZ at different temperatures over time. The values are means±standard deviations of triplicate cultures.

Temperature is one of the most important factors that can change the activity of the enzyme. Cells of strain *E.coli* BL21 harboring pET28a-*phzO* and strain *E.coli* BL21 harboring pET28a were grown at temperatures between 20 and 37°C. Although 20°C is the most suitable temperature for expression of PhzO (Fig. 1), when temperature raised from 20°C to 28°C, the transformation of substrate PCA and the yield of 2-OH-PHZ increased, but when the temperature raised from 28°C to 37°C, the yield of 2-OH-PHZ decrased as shown in Fig. 2.

### PhzO was sufficient to hydroxylate PCA to produce 2-OH-PHZ in the presence of NADP(H) and Fe^3+^

Based on the PCA transformation assay and optimized conditions of HPLC analysis, the IPTG-induced cultures of *E. coli* BL21 harboring pET28a-phzO, converted PCA (0.3–0.5 mg/ml in 5% NaHCO_3_) [14] to 2-OH-PCA after 12 h, while no production of 2-OH-PHZ or 2-OH-PCA occurred in control cultures harboring only the respective pET28a vectors. The production of 2-OH-PHZ increased as that of 2-OH-PCA decreased in the culture of *E. coli* BL21 harboring pET28a-phzO. After 72 h, no 2-OH-PCA could be detected and the yield of 2-OH-PHZ reached a maximum (S1). The result confirmed the previous work done by Delaney *et al*.

We further tested the hydroxyl activity of purified PhzO. Significant activity was detected in the presence of NADP(H) and 1 mM Fe^3+^. The results indicated that PhzO was sufficient to hydroxylate PCA to produce 2-OH-PHZ in the presence of NADP(H) and Fe^3+^ (Table 2). The influence of Fe^3+^ on the levels of 2-OH-PHZ was investigated by adding 1 mM Fe^3+^ to the culture of *E.coli* BL21 harboring pET28a-*phzO*. More 2-OH-PHZ was produced in the cultures containing added Fe^3+^ than in the control groups. The control groups produced about 3 mg/L 2-OH-PHZ, while the groups with 1 mM Fe^3+^ added reached a final concentration of 11 mg/L 2-OH-PHZ after 96h incubation.

2-OH-PHZ production showed a steady increase for additions of PCA of 40–80 mg/L. However, PCA added in excess to the culture inhibited the production of 2-OH-PHZ. Above that amount, at 120 mg/LPCA, the production of 2-OH-PHZ was equivalent to that of 80 mg/L PCA. The quantity of 2-OH-PHZ even dropped when PCA exceeded 160 mg/L.

### Low solubility of 2-OH-PCA in fermentation broth

**Figure 3:**
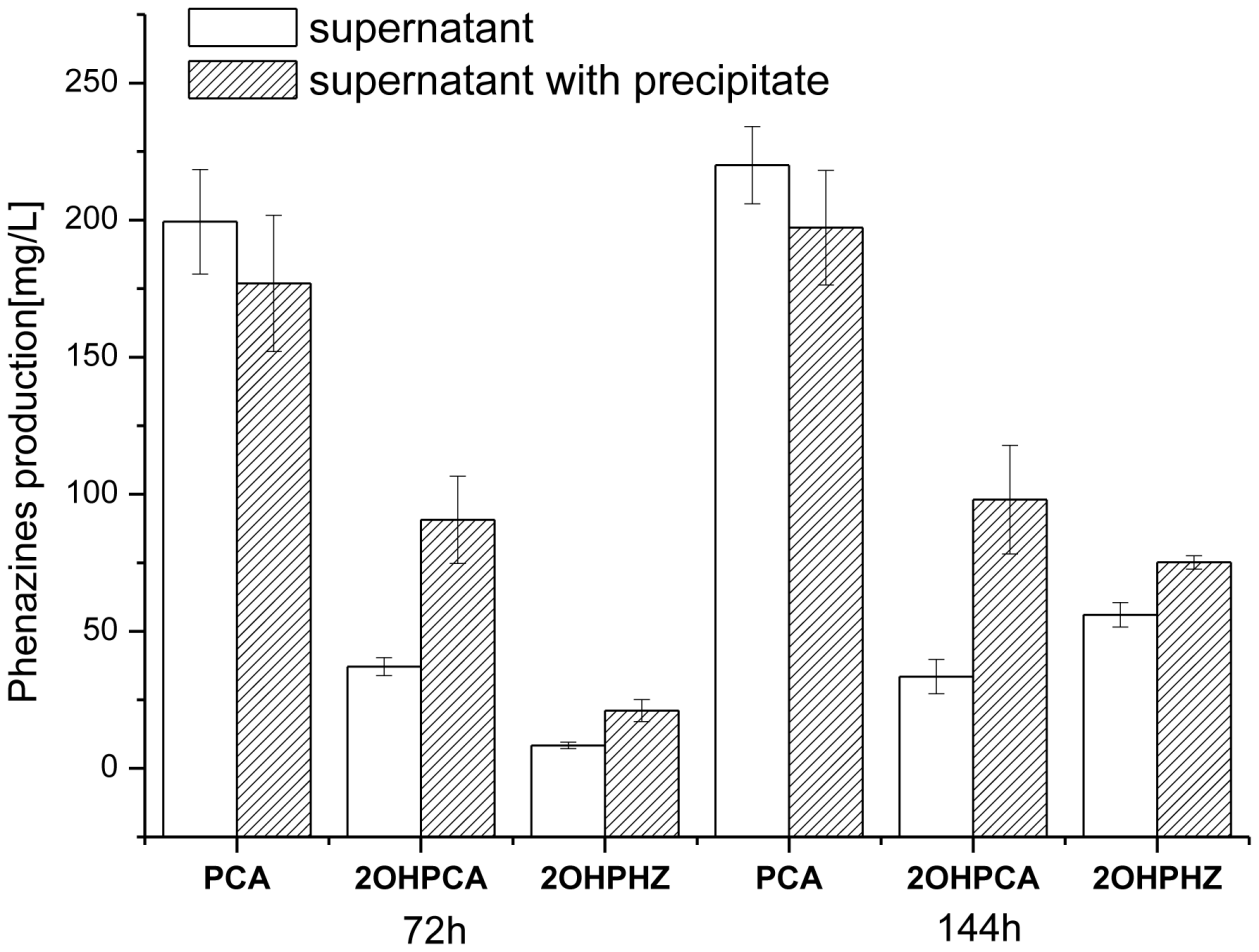
Phenazine derivative production in the supernatant and the precipitate after 72 h and 144 h incubation of GP72AN in KB medium. The values are means±standard deviations of triplicate cultures. Different letters in columns indicate statistically significant differences between treatments (P = 0.05).

*Pseudomonas* GP72AN produced three main phenazines: PCA, 2-OH-PCA and 2-OH-PHZ. Previous work showed that the yield of 2-OH-PCA in *Pseudomonas spp.* GP72AN is relatively low (<50 ppm), only 10–20% of its precursor PCA. Interestingly, red pigment precipitate was observed in the fermentation broth of GP72AN after 72 h incubation, which was determined to be primarily 2-OH-PCA (75%) with lesser amounts of PCA & 2-OH-PHZ as determined by the same HPLC method described previously (Fig. 3). The yield of 2-OH-PCA in the fermentation broth with the red pigment increased to 97±15 mg/L, in comparison with 37±3 mg/L for the supernatant.

### Kinetics at different temperatures

Previous experiments had investigated the stability of 2-OH-PCA in aqueous solution and an organic solvent. The results indicated that 2-OH-PCA was stable in organic solvent even over a week period. However, significant degradation of 2-OH-PCA was observed in aqueous solution while the concentration of 2-OH-PHZ increased over 1 day.

**Figure 4:**
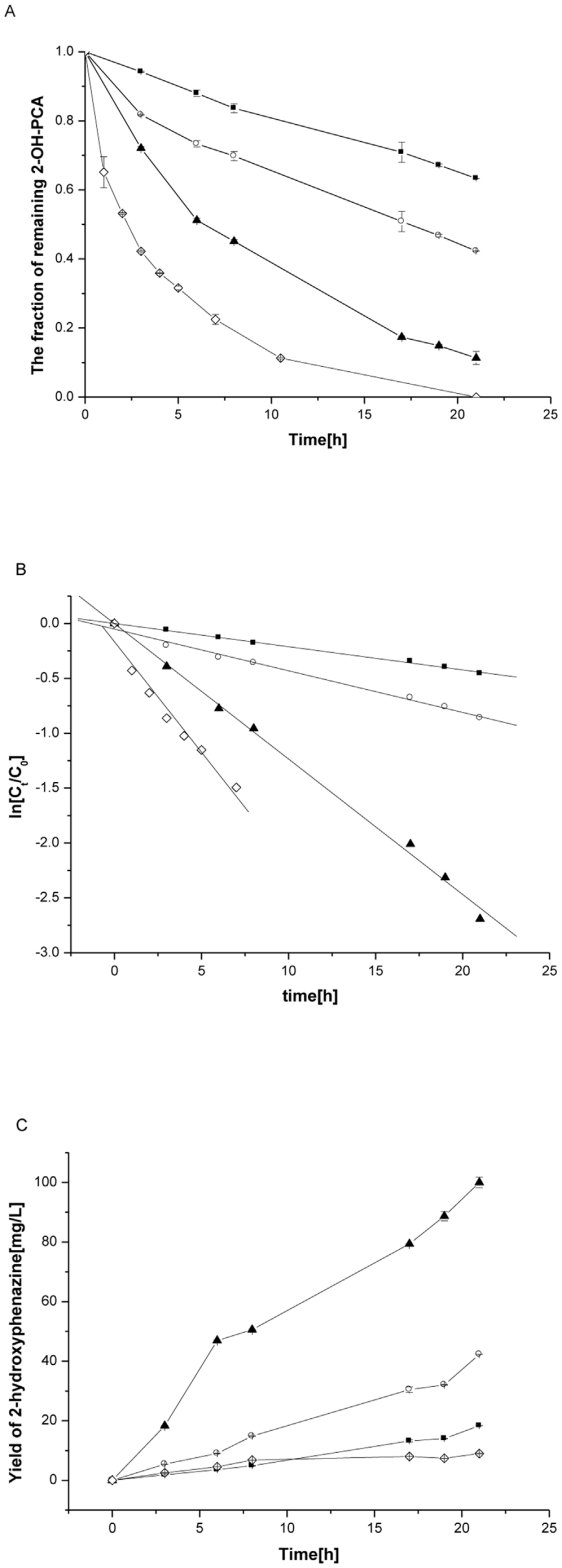
The conversion of 2-OH-PCA to 2-OH-PHZ in phosphate buffer at different temperatures (PBS solution stored in complete darkness). A and B. Degradation of 2-OH-PCA. C. The changes of 2-OH-PHZ over time. *filled square* 28°C; *empty circle* 37°C; *filled triangle* 55°C; *empty diamond* 70°C. Error bars represent standard deviations of data from triplicate samples. The coefficient of variation(standard deviation/mean×100) was below 7% in all cases.



An investigation of the reaction kinetics in aqueous solution at different temperatures revealed that the conversion rate increased with increasing temperature (Fig. 4A). The logarithm concentration rate of 2-OH-PCA (ln [C_t_/C_0_]) versus time plot was linear, and showed that the degradation of 2-OH-PCA follows first-order kinetics. The kinetic equation is represented mathematically as [25]:

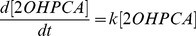
(1)or
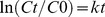
(2)Where k (h*^−^* ^1^) is the rate constant of the first order kinetic reaction, Ct is the concentration of 2-OH-PCA at time t, C_0_ is the initial concentration of 2-OH-PCA. According to Fig. 4B, the first-order rate constant and the corresponding half-lives were calculated and are listed in Table 3.

**Table 3:** Kinetic parameters of 2-OH-PCA degradation at different temperatures.

Temperature °C	Reaction order	Rate constant (h*^−^* ^1^)	R^2^	Half-life (h)
28	1	0.0206	0.9979	33.64
37	1	0.0378	0.9898	18.33
55	1	0.1197	0.9993	5.79
70	1	0.1936	0.9818	3.58

The logarithm of the first-order constant versus the reciprocal of temperature (K) plots were linear and can be expressed by the Arrhenius equation [25]

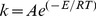
(3)or
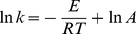
(4)where k (h*^−^* ^1^) is the rate constant of the first order kinetic reaction, T is the temperature in Kelvin (K), E is the activation energy (kJ mol*^−^* ^1^), A is the frequency factor and R is the molar gas constant (8.314 kJ mol*^−^* ^1^).

From Fig. 4B **,** the half-lives of 2-OH-PCA at various temperatures were calculated, and is an important consideration when planning the handling and storage of 2-OH-PCA. Unfortunately, less 2-OH-PHZ than expected was detected at 70°C, as 2-OH-PHZ is unstable at high temperatures (Fig. 4C).

### Kinetics at different pHs

**Figure 5:**
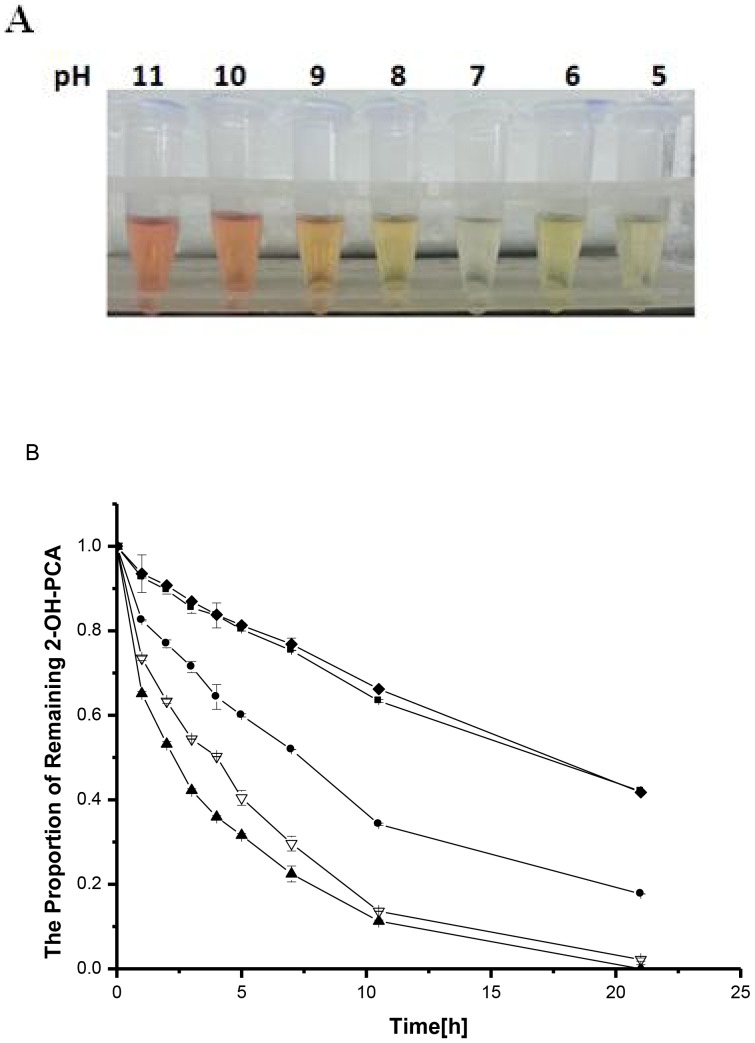
The conversion of 2-OH-PCA to 2-OH-PHZ in phosphate buffer at different pHs (water solution stored at 70°C in complete darkness). A: 2-OH-PCA with phosphate buffer at different pHs. B: Degradation of 2-OH-PCA at different pHs. *filled diamond* pH 11.0; *filled square* pH 3.0; *filled circle* pH 6.0; *empty triangle* pH 9.0; *filled triangle* pH 7.0. Error bars represent standard deviations of data from triplicate samples. The coefficient of variation (standard deviation/mean×100) was below 3% in all cases.

Previous study revealed that 2-OH-PCA was sensitive to changes in pH and it had distinctive coloration depending on its pH environment [14]. To understand the effect of pH on the reaction, the conversion of 2-OH-PCA to 2-OH-PHZ in PBS at pHs 3.0-11.0 was studied (Fig. 5A). The results showed that the optimal pH for the conversion was 7.0. As shown in Fig. 5B, the concentration of 2-OH-PCA was 42%, 17%, 0%, 2%, and 41% of the initial value after 21 h at pH 3.0, 6.0, 7.0, 9.0 and 11.0, respectively, and the first-order rate constant and the corresponding half-lives were calculated and are listed in Table 4.

**Table 4:** Kinetic parameters of 2-OH-PCA degradation at different pH values.

pH	Reaction order	Rate constant (h*^−^* ^1^)	R^2^	Half-life (h)
3	1	0.0407	0.9978	17.03
6	1	0.0813	0.985	8.53
7	1	0.1936	0.9818	3.58
9	1	0.1812	0.9974	3.82
11	1	0.0408	0.9965	16.99

### Photodegradation

**Figure 6:**
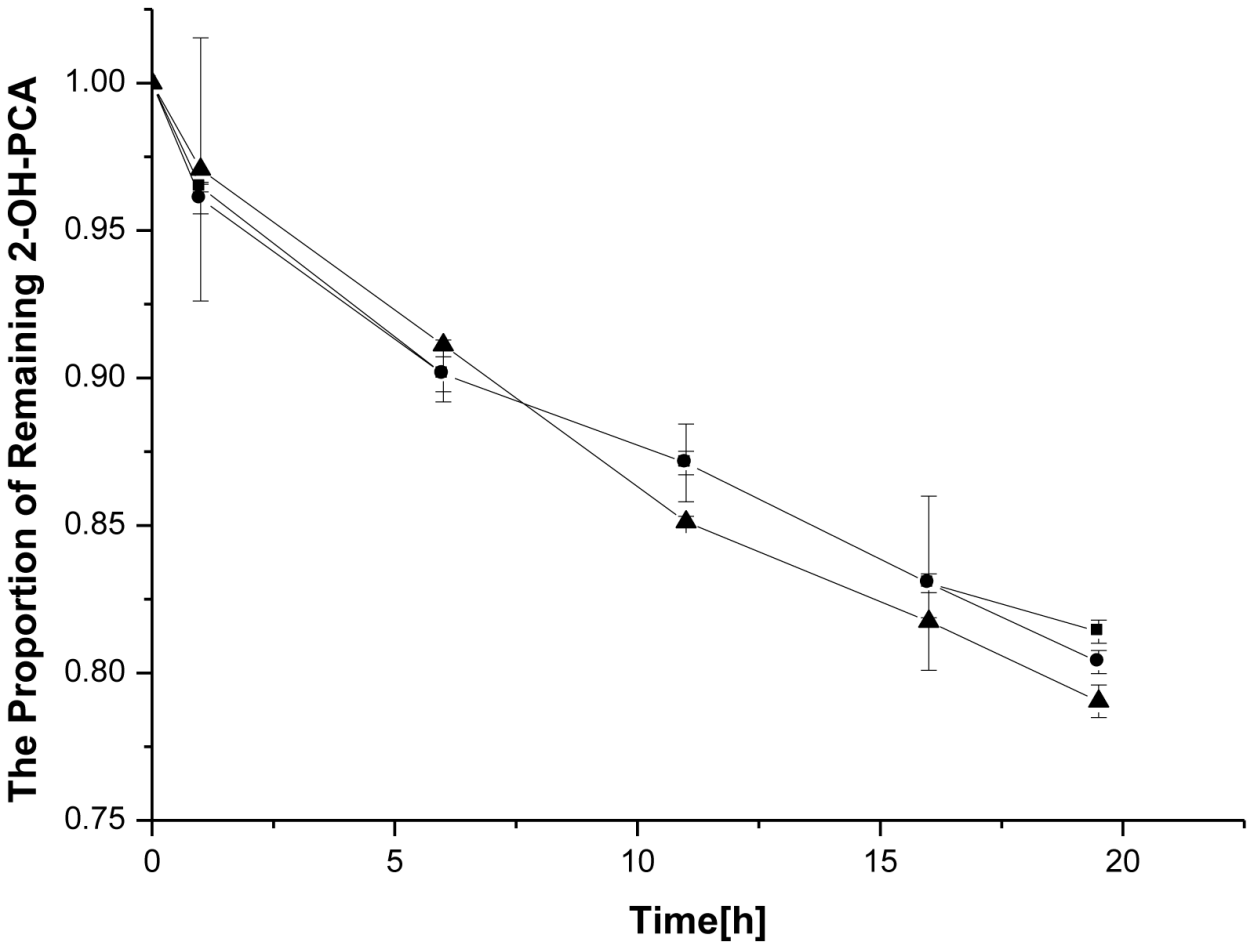
The conversion of 2-OH-PCA to 2-OH-PHZ under different light sources at room temperature. *filled square* dark; *filled circle* fluorescent lamp; *filled triangle* ultraviolet lamp. Error bars represent standard deviations of data from triplicate samples.

Many natural compounds are light sensitive, such as plt, aldicarb, parathion, mecoprop, linuron and chlorpyifos. Previous study in our lab discovered that PCA was light sensitive (data not shown). Here the stability of the conversion of 2-OH-PCA under different lights were studied. An ultraviolet lamp and a fluorescent lamp were used to measure the effects of different irradiation. The results showed that the light has less effect on the conversion of 2-OH-PCA to 2-OH-PHZ in contrast to the conversion of 2-OH-PCA to 2-OH-PHZ in the dark (Fig. 6).

### The conversion of 2-OH-PCA to 2-OH-PHZ in cell extracts of *Pseudomonas chlororaphis and its mutants*

As shown in Table 5, enzymes in cell extracts did not accelerate the reaction of intermediate product 2-OH-PCA that underwent spontaneous decarboxylation to form 2-OH-PHZ. With the activity of enzyme of GP72FN or GP72ON, 2-OH-PHZ accounts for 10% of the total phenazine present after 18h, while 14% of the initial 2-OH-PCA underwent decarboxylation to form 2-OH-PHZ without any enzyme in PBS, according with the results after 48h incubation.

**Table 5:** The influence of enzymes in cells extracts on the conversion of 2-OH-PCA to 2-OH-PHZ.

Strains	Concentration of phenazines (mg/L)					
	0h		18h		48h	
	2-OH-PCA	2-OH-PHZ	2-OH-PCA	2-OH-PHZ	2-OH-PCA	2-OH-PHZ
GP72FN	30	0	24.42±0.94	2.00±0.02	21.42±0.05	3.11±0.95
GP72ON	30	0	26.09±0.91	1.49±0.06	23.12±0.84	3.74±0.01
Control^1^	30	0	25.24±0.75	1.31±0.02	21.42±1.25	3.61±0.32

1 The control group was 30 mg/L 2-OH-PCA in PBS without any enzyme.

The experiment was performed three times and means±SE of triplicate experiments from same culture are plotted.

## Discussion

In recent studies, based on PCA as a precursor, several phenazine biosynthetic related genes were found to play roles in the synthesis of specific phenazine derivatives including *phzH*, *phzM*, *phzS* and *phzO.* PhzH [26] could convert PCA to PCN in *P. aeruginosa* PAO1 while PhzM could catalyze PCA to pyocyanin (PYO) with the help of PhzS [27]. PhzO in *P. chlororaphis* 30–84 and *P. chlororaphis* GP72 was found to hydroxylate PCA to 2-OH-PHZ [11], [14]. Both strains could produce 2-OH-PHZ and PCA, and GP72 and 30–84 were supposed to have similar genomic structures. Based on the genomic data of *P. chlororaphis* GP72 [10], the phenazine biosynthetic genes and regulators have been investigated in an effort to improve the production of phenazines.

As PhzO belongs to the family of flavin-diffusible monooxygenase NAD(P)H-dependent flavoproteins, a lack of NADP (H) will also result in the inactivation of PhzO, which would block the conversion of PCA to 2-OH-PCA. In this study, PhzO was heterologously expressed in *E. coli* BL21, and successfully used to convert free PCA to 2-OH-PHZ confirming and complementing the reaction mechanism of 2-OH-PHZ discovered by Delaney *et al.* [14]. We showed that this conversion was dependent on the presence of sufficient NADP(H) and Fe^3+^. However, the hydroxylation of PCA was inefficient, as there was PCA remaining even after 72 h incubation. A number of studies have shown that 2-hydroxyphenazines have stronger antibiotic activity than PCA toward some pathogens [14], [28], but the low yield of 2-OH-PHZ has limited its applications. The low yield of 2-OH-PHZ is attributed to the low conversion rate of PCA to 2-OH-PHZ (10–20%) [11].

One reason for the low hydroxylation efficiency is the low PhzO yield. We analyzed the soluble and insoluble fractions of PhzO, and found that the expressed PhzO protein was largely obtained in the insoluble inclusion bodies, thus reduced the overall yield of active enzyme (Fig. 1), and reduced the transformation of PCA.

Another reason for the low 2-OH-PHZ yield is the low solubility of 2-OH-PCA. In this study, the intermediate 2-OH-PCA was observed to accumulate as a red pigmented precipitate in the fermentation broth, and could be easily isolated and purified. The fact that 2-OH-PCA can be completely converted to 2-OH-PHZ coupled with the observation that solid 2-OH-PCA was stable over a long period supports the hypothesis that the low conversion of PCA to 2-OH-PHZ is partly due to the low solubility of 2-OH-PCA in the fermentation broth. The precipitation of 2-OH-PCA slowed the conversion from 2-OH-PCA to 2-OH-PHZ and thus, reduced the yield of 2-OH-PHZ.

Iron is an important element that regulates some secondary metabolisms of *Pseudomonas* [29], [30]. In this work, Fe^3+^ enhances PhzO enzyme activity,indicating that the ratio of conversion from PCA to 2-OH-PHZ could be enhanced by adding Fe^3+^, and the latter experiment in vivo confirmed that assumption based on the result that the yield of 2-OH-PHZ doubled by adding 1 mM Fe^3+^. One hypothesis may be proposed to explain this result. PCA has been reported that can reduce Fe^3+^ reduction [31]. Thus, the addition of Fe^3+^ may oxidize PCA and accelerated the reaction that PCA converts to 2-OH-PCA. Work on the regulatory mechanism of Fe^3+^ on 2-hydroxyphenazines biosynthesis is now in progress.

The quantity of 2-OH-PHZ would be expected to increase when PCA accumulated as it was derived from PCA. No more 2-OH-PHZ was produced in the cultures with the addition of PCA of 80–120 mg/L, indicating that saturation of enzyme PhzO might be occurring under the current culture conditions and fermentation process. Above that amount, for example, at 280 mg/L PCA, the production of 2-OH-PHZ was less than that of 80 mg/L, indicating that substrate inhibition of enzyme PhzO might occurred above 120 mg/L PCA.

The results of our study confirmed the reaction mechanism proposed by Delaney *et al.* that 2-OH-PCA is spontaneously converted to 2-OH-PHZ in a pH dependent manner [14]. Here it is determined that the conversion of 2-OH-PCA to 2-OH-PHZ followed first order kinetics at different pH values. Based on these results, we assumed that the ratio of conversion from 2-OH-PCA to 2-OH-PHZ is dependent on the different ionic forms of 2-OH-PCA which vary depending on the pH environment.

The ratio of the conversion from 2-OH-PCA to 2-OH-PHZ increased with the reaction temperature when it was below 55°C, but decreased at temperatures above 70°C. Increasing temperature causes 2-OH-PCA degradation along with a decrease in 2-OH-PHZ.

**Figure 7:**
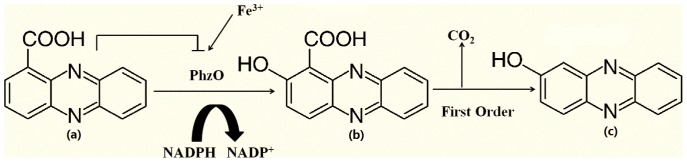
Complemented mechanism of the conversion of PCA to 2-OH-PHZ: PhzO hydroxylates PCA to 2-OH-PCA in the presence of NADP(H), Fe^3+^ enhanced the activity of PhzO. Subsequently, the intermediate product 2-OH-PCA spontaneously decarboxylates to form 2-OH-PHZ followed first-order reaction kinetics. The chemical structure of phenazine-1-carboxylic acid (PCA) (a), 2-hydroxy phenazine-1-carboxylic acid (2-OH-PCA) (b) and 2-hydroxyphenazine (2-OH-PHZ) (c). Arrows indicate positive direction of reaction, lines with flat ends indicate negative regulation.

In this study, we discovered that PhzO was sufficient to hydroxylate PCA to produce 2-OH-PHZparticularly in the presence of sufficient NADP(H) and Fe^3+^, advancing the current knowledge of the biosynthesis pathway of 2-OH-PHZ (Fig. 7). Further we find out that Fe^3+^ enhanced the conversion from PCA to 2-OH-PHZ and the optimum temperature for the conversion was 28°C. Significantly, the substrate inhibition of enzyme PhzO was investigated. Then a kinetic model was developed to study the conversion kinetics of 2-OH-PHZ. The results showed that temperature and pH values of solutions were the factors that had a strong influence on the conversion of 2-OH-PCA to 2-OH-PHZ. Degradation of 2-OH-PCA followed first-order reaction kinetics, and the rate constant increased with increased temperature but decreased when pH below or above 7.0. In addition, we confirmed that the intermediate product 2-OH-PCA underwent spontaneous decarboxylation to form 2-OH-PHZ and this reaction was not accelerated by any enzyme in *Pseudomonas* extracts such like PhzC, PhzD and PhzE. This study advances the current understanding of the biosynthetic pathway responsible for the production of 2-OH-PHZ by providing a detailed description of the reaction kinetics for the biocatalytic conversion of PCA to 2-OH-PHZ. This information should provide for improvements in 2-OH-PHZ yield and wide application of 2-OH-PHZ as biopesticiede.

## Supporting Information

Figure S1 **The expression profile of protein PhzO in** ***E. coli*** **BL21. (A) The expression of PhzO in** ***E. coli*** **BL21.** Lane 1: Whole cell lysate of BL21 harboring pET28a; M: Premixed Protein Marker (Low); Lane 2: Whole cell lysate of BL21 harboring pET28a-phzO. (B) The purification of PhzO. M: Premixed Protein Marker (High); Lane1: Whole cell lysate of BL21 harboring pET28a; Lane 2: PhzO purified by Ni^2+^-nitrilotriacetic acid chromatography. The gel was loaded with 5 ng of purified PhzO (lane 2).
(TIF)

Figure S2 (A): HPLC analysis of the conversion of PCA to 2-OH-PHZ in *E. coli* BL21 in a PCA transformation assay. (a) Only PCA was detected from cultures of BL21 harboring pET28a after incubation for 72 h; (b) PCA, 2-OH-PCA and 2-OH-PHZ were detected from cultures of BL21 harboring pET28a-phzO after 12 h; (c) PCA and 2-OH-PHZ were detected from cultures of BL21 harboring pET28a-phzO after 72 h; (d) BL21 harboring pET28a without the addition of PCA (B): production of pigments in LB medium after 72 h incubation: (a) E. coli BL21 harboring pET28a; (b) E. coli BL21 harboring pET28a-phzO.
(TIF)
