# Supplementary figures and images for: Reaction Kinetics for the Biocatalytic Conversion of Phenazine-1-Carboxylic Acid to 2-Hydroxyphenazine
Source: PLoS One. 2014 Jun 6;9(6):e98537. doi: 10.1371/journal.pone.0098537 (PMC4048165; doi:10.1371/journal.pone.0098537)

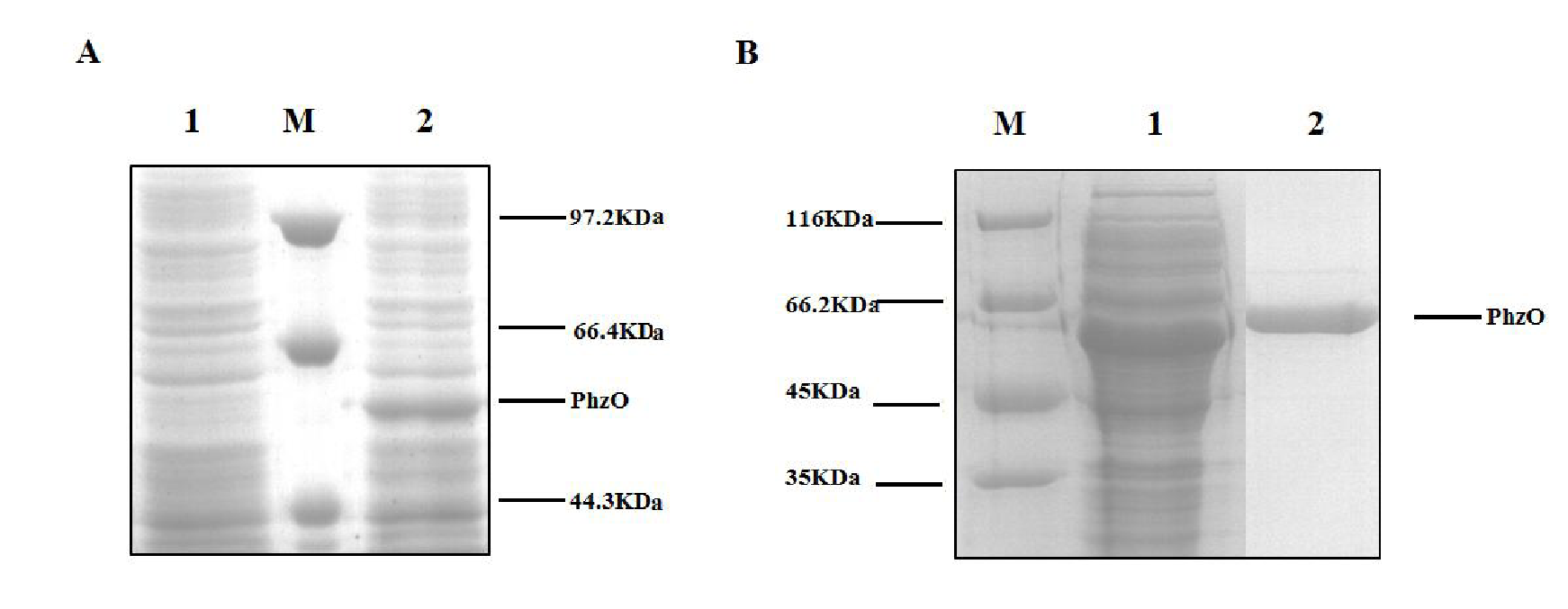

Supplement: Figure S1 — The expression profile of protein PhzO in E. coli BL21. (A) The expression of PhzO in E. coli BL21. Lane 1: Whole cell lysate of BL21 harboring pET28a; M: Premixed Protein Marker (Low); Lane 2: Whole cell lysate of BL21 harboring pET28a-phzO. (B) The purification of PhzO. M: Premixed Protein Marker (High); Lane1: Whole cell lysate of BL21 harboring pET28a; Lane 2: PhzO purified by Ni2+-nitrilotriacetic acid chromatography. The gel was loaded with 5 ng of purified PhzO (lane 2). (TIF) [file pone.0098537.s001.tif]

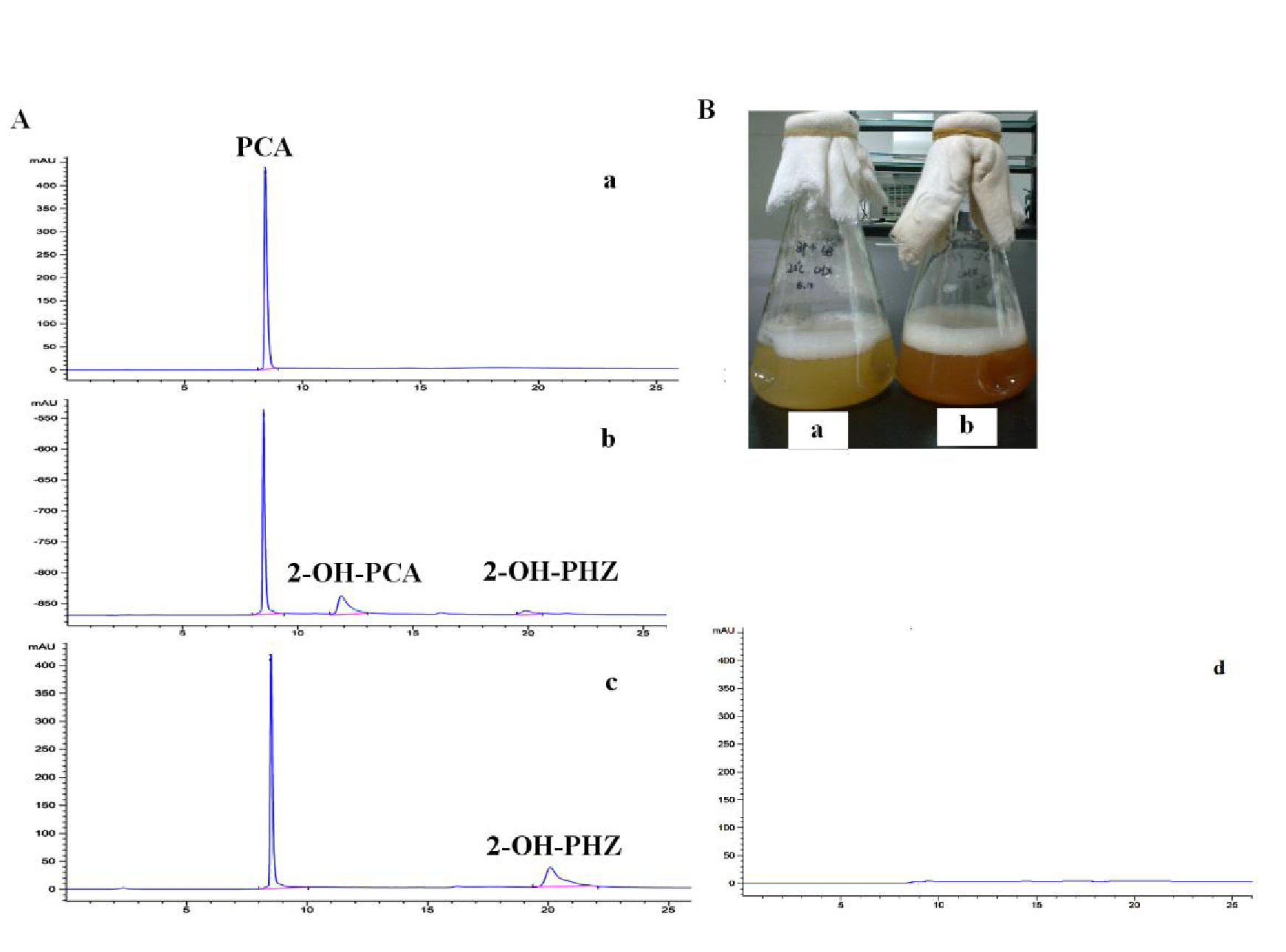

Supplement: Figure S2 — (A): HPLC analysis of the conversion of PCA to 2-OH-PHZ in E. coli BL21 in a PCA transformation assay. (a) Only PCA was detected from cultures of BL21 harboring pET28a after incubation for 72 h; (b) PCA, 2-OH-PCA and 2-OH-PHZ were detected from cultures of BL21 harboring pET28a-phzO after 12 h; (c) PCA and 2-OH-PHZ were detected from cultures of BL21 harboring pET28a-phzO after 72 h; (d) BL21 harboring pET28a without the addition of PCA (B): production of pigments in LB medium after 72 h incubation: (a) E. coli BL21 harboring pET28a; (b) E. coli BL21 harboring pET28a-phzO. (TIF) [file pone.0098537.s002.tif]
